# Analysis of flow rate of continuous bladder irrigation according to the height of the irrigation infusion set

**DOI:** 10.1038/s41598-023-47198-2

**Published:** 2023-11-12

**Authors:** Boeun Yang, Jeongwon Han

**Affiliations:** College of Nursing Science, Kyung Hee University, Seoul, Republic of Korea

**Keywords:** Patient education, Bladder

## Abstract

This is a control volume analysis to examine the flow rate of irrigation fluid according to the size of indwelling catheter and the height of the fluid bag in consideration of the temperature of irrigation fluid and intra-bladder pressure during continuous bladder irrigation. In case of minimum bladder pressure with room temperature, the flow rates were − 0.045 to 0.993 cc/sec for 18Fr, − 0.053 to 1.176 cc/sec for 20Fr, − 0.055 to 1.227 cc/sec for 22Fr, and − 0.055 to 1.243 cc/sec for 24Fr. In case of maximum bladder pressure with room temperature, the flow rates were − 0.180 to 0.868 cc/sec for 18Fr, − 0.212 to 1.028 cc/sec for 20Fr, − 0.220 to 1.072 cc/sec for 22Fr, and − 0.223 to 1.086 cc/sec for 24Fr. In case of minimum bladder pressure with cold fluid, the flow rates were − 0.028 to 0.365 cc/sec for 18Fr, − 0.033 to 0.749 cc/sec for 20Fr, − 0.034 to 0.781 cc/sec for 22Fr, and − 0.035 to 0.791 cc/sec for 24Fr. In case of maximum bladder pressure with cold fluid, the flow rates were − 0.112 to 0.553 cc/sec for 18Fr, − 0.131 to 0.653 cc/sec for 20Fr, − 0.137 to 0.681 cc/sec for 22Fr, and − 0.139 to 0.689 cc/sec for 24Fr. This study is significant in that it utilized a fluid dynamics approach to provide basic data for continuous bladder wash care. Through the findings of this study, nurses can plan the exchange time of irrigation fluid and the pattern of urinary drainage when performing continuous bladder irrigation. It is also inferred that there may be an advantage in not having to calculate additional material costs for using an infusion pump for patients by determining the hourly injection rate of irrigation fluid based on the height of the infusion set's drop chamber.

## Introduction

With South Korea being an aged society, the incidence of aging-related urological conditions, such as benign prostate hyperplasia (BPH) and urinary incontinence^1^, has increased. The main symptoms of urological diseases include voiding disorder or bladder hemorrhage, and bladder hemorrhage should be monitored, particularly after surgeries or procedures such as transurethral resection of the prostate (TURP) and open prostatectomy^2–4^. Patients who are anticipated to develop bladder hemorrhage or those who have developed it undergo intermittent or continuous bladder irrigation (CBI) through indwelling catheters to prevent and remove thrombi in the bladder^3, 5, 6^.

Nurses who provide care to patients with an indwelling catheter replace indwelling catheters, irrigate bladders, train bladders, and provide perineal care, with care related to bladder irrigation accounting for approximately 35% of nursing care provided to urological inpatients^7^. To irrigate the bladder effectively, the nurse considers a variety of factors, such as irrigation fluid, size of the indwelling catheter, and height of irrigation fluid^8^. Normal saline (NS) at room temperature is generally used for bladder irrigation to prevent bladder spasms and water intoxication^6, 9^, and cold saline (4 °C) may be used to reduce hemorrhage through vasoconstriction in cases of severe bladder hemorrhage^10^. CBI is generally performed using a three-lumen catheter^3, 11^, with an 18Fr catheter for patients without severe hematuria and a 22Fr catheter for those expected to develop severe hematuria^2^.

The flow rate for CBI should be considered to prevent and remove bladder thrombosis^12^. During CBI, the nurse checks the continuous removal of irrigation fluid to maintain the color of urine at soft pink or colorless^13^, and if hematuria increases or massive thrombosis is observed, the flow rate is adjusted by consulting with a physician^5^. The flow rate refers to the volume of fluid flowing per unit time, and most bladder irrigation guidelines recommend increasing the flow rate in cases of massive thrombosis observed in the drained urine and increasing the flow rate by elevating the fluid bag^9^. However, the height of the fluid bag and flow rate are not specified in these guidelines, which hinders nurses from accurately adjusting the flow rate, and many nurses only passively monitor the flow rate or estimate it based on their experiences^14^.

Moreover, Reichelt et al.^14^ reported that approximately 50% of urological inpatients undergo bladder irrigation, showing that bladder irrigation accounts for a substantial percentage of nurses’ work. Furthermore, critical patients are at risk of developing hemodynamic instability during bladder irrigation; therefore, the nurse pays considerable attention to adjusting the flow rate and uses medical equipment such as an infusion pump or pressure infuser bag to lessen their burden of adjusting the flow rate^3^. However, the use of infusion pumps or pressure infuser bags for urological procedures is only reimbursed in limited conditions in which the flow rate is adjusted to secure the field of view when removing ureter stones. Hence, using infusion pumps for adjusting the flow rate during a regular bladder irrigation is not favorable in terms of cost for the hospital as well as patients^15^.

Most past studies on the flow rate during CBI have analyzed the variations in flow rate by the size of indwelling catheter. Chang et al.^12^ presented the height of fluid bag as a predictor of flow rate and measured flow rates by altering the height of the fluid bag by 20 cm from the height of the bladder to 140 cm. The study found that there are no changes in the flow rate from 80 cm. However, one limitation of the study by Chang et al.^12^ is that the authors used Bernoulli’s equation, which did not consider friction, despite the predominant flow within the tube. Moreover, Bernoulli’s equation must be used for a steady flow, but the study mentioned above included unstable flow in the area connecting the fluid bag to the irrigation infusion set. Additionally, different irrigation fluids are used in clinical practice depending on the patient, and the nurse performs bladder irrigation in consideration of various factors such as the temperature of the fluid and intra-bladder pressure. Nevertheless, previous studies have not reflected these clinical situations. Braasch et al.^16^ emphasized the importance of the diameter of indwelling catheters in increasing the flow rate and experimented with the fluid bag fixed at 80 cm. However, they did not present evidence of their choice of fluid bag height. Mohammadi^9^ also analyzed flow rates using Bernoulli’s equation but did not consider friction in their analysis.

Studies that utilize fluid mechanics present practical evidence and are used for reference in several fields, such as automotive, aerospace, manufacturing, construction, medicine, and pharmacy, as well as for the implementation of social distancing measures^17–21^. Flow rate and pressure were studied in nursing in relation to wound irrigation^22^. Analyzing the variations in the flow rate according to the height of the fluid bag during CBI helps reduce nurses’ subjective judgments and specify the flow rate recommendations in guidelines, which allows nurses to perform bladder irrigation more effectively in clinical practice. In this context, this study aims to analyze the flow rate according to the height of the fluid bag for different indwelling catheter sizes, in consideration of fluid temperature and intra-bladder pressure, eventually presenting foundational data for flow rate adjustment during CBI.

## Results

The flow rates were analyzed for different sizes of indwelling catheters and heights of irrigation infusion sets using room-temperature and minimum bladder pressure at resting supine position. At all sizes, the lowest flow rate was at a height of 0 cm, and the highest was at 120 cm. A negative flow rate indicates that irrigation fluid is regurgitating due to a stronger force from the bladder to the irrigation infusion set compared with the force from the irrigation infusion set to the bladder because of intra-bladder pressure.

### Analysis of flow rate with room temperature fluid (22 °C 0.9% NS) by the height of the irrigation infusion set

**Figure 1:**
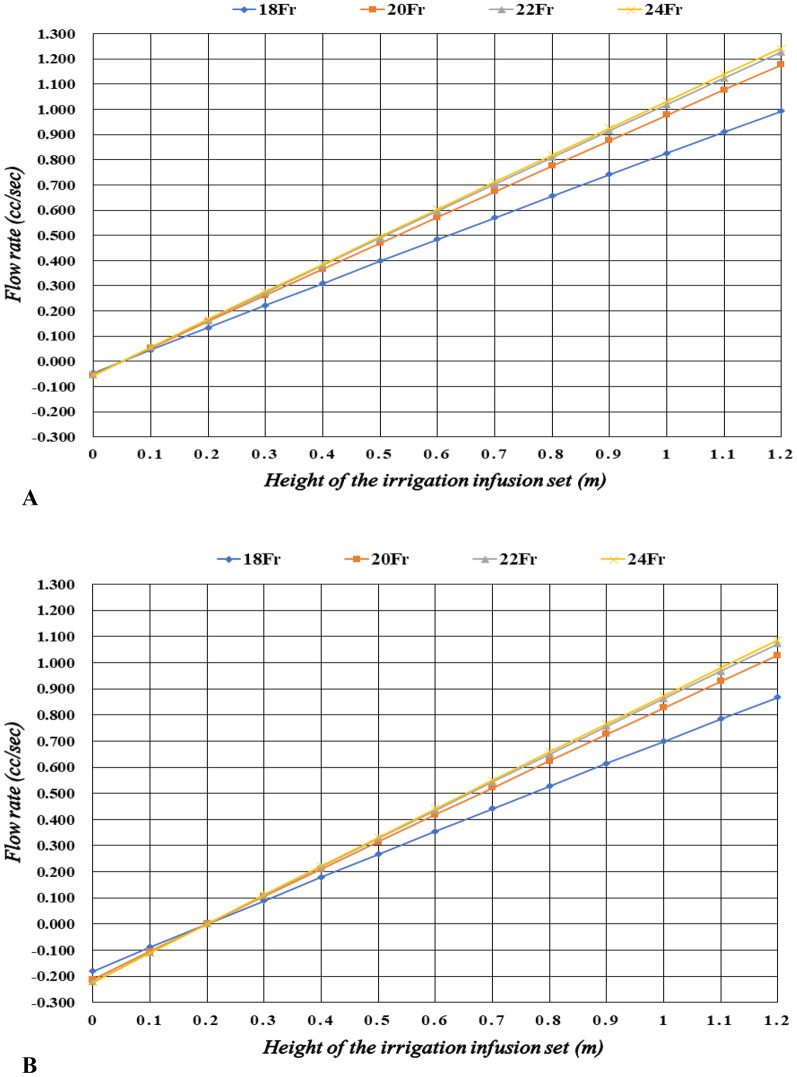
(**A**) The flow rates for different sized of indwelling catheters with room temperature fluid (22 °C 0.9% NS) and minimum bladder pressure (5 cmH_2_O) by the height of the irrigation infusion set. (**B**) The flow rates for different sized of indwelling catheters with room temperature fluid (22 °C 0.9% NS) and maximum bladder pressure (20 cmH_2_O) by the height of the irrigation infusion set.

In case of minimum bladder pressure (5 cmH_2_O), the flow rates were − 0.045 to 0.993 cc/sec for 18Fr, − 0.053 to 1.176 cc/sec for 20Fr, − 0.055 to 1.227 cc/sec for 22Fr, and − 0.055 to 1.243 cc/sec for 24Fr (Table 1) (Fig. 1A). In case of maximum bladder pressure (20 cmH_2_O), the flow rates were − 0.180 to 0.868 cc/sec for 18Fr, − 0.212 to 1.028 cc/sec for 20Fr, − 0.220 to 1.072 cc/sec for 22Fr, and − 0.223 to 1.086 cc/sec for 24Fr (Table 2; Fig. 1B).

**Table 1:** The flow rates for room temperature fluid and minimum bladder pressure by the height of the irrigation infusion set.

Height (m)	Flow rate (cc/s)			
	18Fr	20Fr	22Fr	24Fr
0	− 0.045	− 0.053	− 0.055	− 0.055
0.1	0.045	0.053	0.055	0.056
0.2	0.134	0.158	0.165	0.167
0.3	0.222	0.262	0.273	0.277
0.4	0.310	0.366	0.382	0.386
0.5	0.398	0.469	0.489	0.495
0.6	0.484	0.572	0.596	0.604
0.7	0.570	0.674	0.703	0.712
0.8	0.656	0.776	0.809	0.819
0.9	0.741	0.877	0.914	0.926
1.0	0.826	0.977	1.019	1.032
1.1	0.910	1.077	1.123	1.138
1.2	0.993	1.176	1.227	1.243

**Table 2:** The flow rates for room temperature fluid and maximum bladder pressure by the height of the irrigation infusion set.

Height (m)	Flow rate (cc/s)			
	18Fr	20Fr	22Fr	24Fr
0	− 0.180	− 0.212	− 0.220	− 0.223
0.1	− 0.089	− 0.105	− 0.109	− 0.111
0.2	0.001	0.001	0.001	0.001
0.3	0.090	0.106	0.111	0.112
0.4	0.179	0.211	0.220	0.223
0.5	0.267	0.315	0.328	0.332
0.6	0.355	0.419	0.436	0.442
0.7	0.442	0.521	0.544	0.550
0.8	0.528	0.624	0.650	0.659
0.9	0.614	0.726	0.757	0.766
1.0	0.699	0.827	0.862	0.873
1.1	0.784	0.928	0.967	0.980
1.2	0.868	1.028	1.072	1.086

### Analysis of flow rate with cold fluid (4 °C 0.9% NS) by the height of the irrigation infusion set

**Figure 2:**
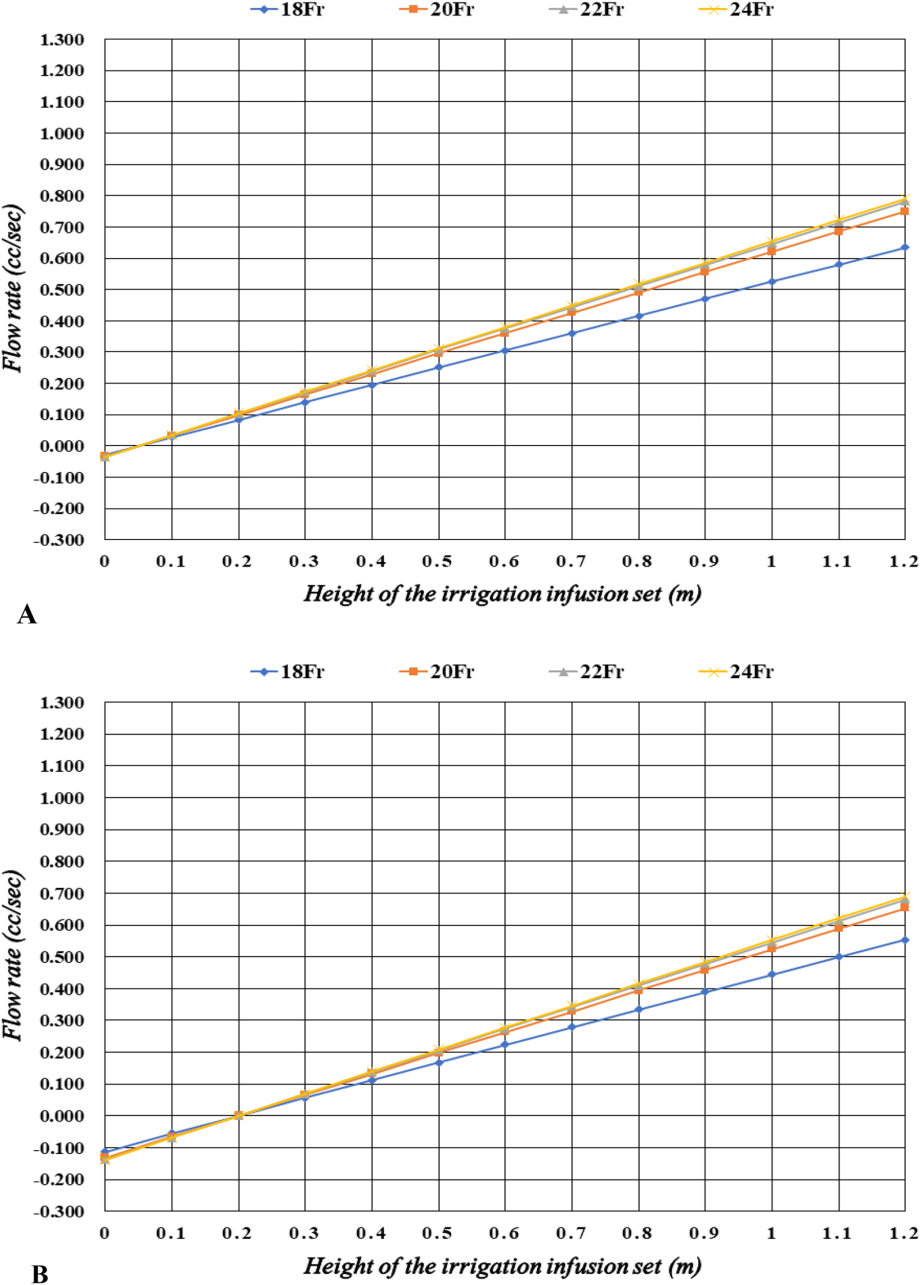
(**A**) The flow rates for different sized of indwelling catheters with cold fluid (4 °C 0.9% NS) and minimum bladder pressure (5 cmH_2_O) by the height of the irrigation infusion set. (**B**) The flow rates for different sized of indwelling catheters with cold fluid (4 °C 0.9% NS) and maximum bladder pressure (20 cmH_2_O) by the height of the irrigation infusion set.

In case of minimum bladder pressure (5 cmH_2_O), the flow rates were − 0.028 to 0.365 cc/sec for 18Fr, − 0.033 to 0.749 cc/sec for 20Fr, − 0.034 to 0.781 cc/sec for 22Fr, and − 0.035 to 0.791 cc/sec for 24Fr (Table 3) (Fig. 2A). In case of maximum bladder pressure (20 cmH_2_O), the flow rates were − 0.112 to 0.553 cc/sec for 18Fr, − 0.131 to 0.653 cc/sec for 20Fr, − 0.137 to 0.681 cc/sec for 22Fr, and − 0.139 to 0.689 cc/sec for 24Fr (Table 4; Fig. 2B).

**Table 3:** The flow rates for cold fluid and minimum bladder pressure by the height of the irrigation infusion set.

Height (m)	Flow rate (cc/s)			
	18Fr	20Fr	22Fr	24Fr
0	− 0.028	− 0.033	− 0.034	− 0.035
0.1	0.028	0.033	0.035	0.035
0.2	0.084	0.099	0.103	0.104
0.3	0.140	0.165	0.172	0.174
0.4	0.195	0.230	0.240	0.243
0.5	0.251	0.296	0.308	0.312
0.6	0.306	0.361	0.376	0.381
0.7	0.361	0.426	0.444	0.449
0.8	0.416	0.491	0.511	0.518
0.9	0.471	0.556	0.579	0.586
1.0	0.526	0.620	0.646	0.655
1.1	0.580	0.685	0.714	0.723
1.2	0.635	0.749	0.781	0.791

**Table 4:** The flow rates for cold fluid and maximum bladder pressure by the height of the irrigation infusion set.

Height (m)	Flow rate (cc/s)			
	18Fr	20Fr	22Fr	24Fr
0	− 0.112	− 0.131	− 0.137	− 0.139
0.1	− 0.055	− 0.065	− 0.068	− 0.069
0.2	0.001	0.001	0.001	0.001
0.3	0.057	0.067	0.069	0.070
0.4	0.112	0.132	0.138	0.140
0.5	0.168	0.198	0.206	0.209
0.6	0.224	0.264	0.274	0.278
0.7	0.279	0.329	0.343	0.347
0.8	0.334	0.394	0.410	0.416
0.9	0.389	0.459	0.478	0.484
1.0	0.444	0.524	0.546	0.553
1.1	0.499	0.589	0.613	0.621
1.2	0.553	0.653	0.681	0.689

### Comparison of flow rate according to the size of indwelling catheter and height of irrigation infusion set

At all sizes of the indwelling catheter used in this study (18–24Fr), the flow rate significantly increased with increasing height of irrigation infusion set when room-temperature fluid (22 °C 0.9% NS) was used, compared with cold fluid (4 °C 0.9% NS). Furthermore, at all sizes of the indwelling catheter, the flow rate significantly increased with increasing height of the irrigation infusion set in the lower bladder pressure condition (Supplement 1; Tables 1, 2, 3, 4). For the comparison of flow rate increase with respect to height, we compared the average flow rate increase when irrigation infusion set's height increased from 0.2 m to 1.2 m. The reason for calculating the average flow rate increase from 0.2 m is that irrigation infusion set’s height does not backflow in all analysis cases, and in the real clinical environment, a height of less than 0.2 m is practically impossible. At room temperature and low pressure, the average flow rate increase per unit meter of height for 18Fr is 0.859 cc/sec m, for 20Fr is 1.018 cc/sec m, for 22Fr is 1.062 cc/sec m, and for 24Fr is 1.076 cc/sec m. At room temperature and high pressure, the average flow rate increase per unit meter of height for 18Fr is 0.867 cc/sec m, for 20Fr is 1.027 cc/sec m, for 22Fr is 1.071 cc/sec m, and for 24Fr is 1.085 cc/sec m. At cold temperature and low pressure, the average flow rate increase per unit meter of height for 18Fr is 0.551 cc/sec m, for 20Fr is 0.650 cc/sec m, for 22Fr is 0.678 cc/sec m, and for 24Fr is 0.687 cc/sec m. At cold temperature and high pressure, the average flow rate increase per unit meter of height for 18Fr is 0.552 cc/sec m, for 20Fr is 0.652 cc/sec m, for 22Fr is 0.680 cc/sec m, and for 24Fr is 0.688 cc/sec m.

### Validity of the assumption of laminar flow

In terms of the assumption of laminar flow of the irrigation fluid based on the size of indwelling catheter and height of irrigation infusion set for 22 °C 0.9% NS and minimum (5 cmH_2_O) and maximum (20 cmH_2_O) bladder pressures at resting supine position, the results showed that theReynolds number from the point at ½ height of the drip chamber to the end of the irrigation infusion set (points 1 and 2 of the analysis target) were all below 2100. This indicates that the assumption of laminar flow is satisfied. Furthermore, the Reynolds number from the end of the irrigation infusion set to that of the indwelling catheter in the same conditions (points 2 and 3 of the analysis target) were all below 2100, indicating that the assumption of laminar flow is satisfied.

## Discussion

This study analyzed the flow rate of irrigation fluid based on the height of the irrigation infusion set for different sizes of indwelling catheters, considering the temperature of the irrigation fluid and the bladder pressure during CBI using the friction-adjusted Bernoulli equation. The main findings are discussed below.

First, when the temperature of the irrigation fluid, bladder pressure, and height of the irrigation infusion set are held constant, the flow rate increases with the increasing diameter of the indwelling catheter. These results are consistent with previous findings^2, 11, 16, 23^ showing a positive relationship between catheter size and flow rate, which enables the effective elimination of urine, including thrombosis in the bladder. Based on the friction-adjusted Bernoulli's equation used in this study, the flow rate of the irrigation fluid entering the bladder is directly proportional to the diameter of the indwelling catheter.

Second, under the same intra-bladder pressure, size of indwelling catheter, and height of the fluid bag, the flow rate into the bladder is greater with room-temperature NS than cold saline. Most past studies^9, 11, 12, 24^ have calculated the flow rate during CBI using room-temperature NS. However, given that irrigation fluids at varying temperatures are used in clinical practice depending on the patient’s state, it is necessary to examine flow rates when using irrigation fluids at varying temperatures. As the viscosity of the fluid plays a role in fluid flow, and lower temperatures increase the viscosity of the irrigation fluid, our results suggest that the flow rate decreases when using cold saline due to increased viscosity and consequently increased friction within the irrigation infusion set. Consequently, a decrease in temperature results in increased frictional forces within the irrigation infusion set. These findings suggest that while using cold saline during CBI, as recommended in relevant guidelines, may help reduce bleeding by stimulating vasoconstriction^10^, it is important to consider the potential trade-off of a decreased flow rate, which may prolong the duration of bladder irrigation. In clinical practice, CBI is performed using cold 0.9% NS in cases of severe bladder hemorrhage to stimulate vasoconstriction of the bladder mucosa and reduce thrombus formation^10^, however, it may be more effective to utilize 0.9% NS at room temperature to increase the irrigation flow rate into the bladder, if removing clots from bleeding is more important^14, 24, 25^.

Third, with the same temperature of the irrigation fluid, size of indwelling catheter, and height of the fluid bag, the flow rate into the bladder is greater with lower intra-bladder pressure. If the purpose of CBI is to eliminate blood clots and urine in the bladder, nurses should consider the patient’s bladder pressure as well as both the size of the indwelling catheter and the height of the irrigation infusion set to facilitate more efficient delivery of the irrigation fluid. Intra-bladder pressure can vary depending on factors such as bladder sensitivity, compliance, stability of the detrusor muscle, maximum bladder capacity, patient position, and bladder and urological conditions such as prostatic obstruction or neurogenic bladder^26, 27^. Therefore, it is important for nursing guidelines related to CBI to incorporate various bladder pressure scenarios and establish a minimum height for the irrigation infusion set accordingly.

Fourth, increasing the height of the irrigation infusion set led to a higher flow rate of the irrigation fluid into the bladder, even when considering the temperature of the fluid, bladder pressure, and catheter size. These results align with Chang et al.^12^, who observe a continuous increase in flow rate up to a height of 80 cm, but differ from their report of a decrease in infusion rate beyond that height. Notably, our study revealed that when the irrigation infusion set height matched that of the bladder or was below 10 cm, the irrigation fluid refluxed from the bladder towards the catheter, which was not reported in previous studies^9, 11, 24^. This discrepancy can be attributed to previous studies using model bladders and setting the bladder pressure to atmospheric pressure, instead of considering the actual bladder pressure in humans, when calculating or experimenting with bladder irrigation flow rate. Consequently, increasing the height of the irrigation infusion set can be an effective approach for nurses to enhance the flow rate of the irrigation fluid, and the duration of irrigation can be estimated based on our findings, highlighting measures to promote efficient work time management.

Fifth, the Reynolds number was calculated to ensure the validity of the assumption of laminar flow during CBI based on the height of the irrigation infusion set; the Reynolds number was below 2100 for all areas of the target of analysis, thereby confirming laminar flow. This finding confirms that the flow rate of CBI using 0.9% NS can be easily calculated using Bernoulli’s equation that considers friction. The flow rates of different types of irrigation fluids for CBI can also be calculated using this equation based on fluid density and viscosity, length and inner diameter of irrigation infusion set, length and inner diameter of indwelling catheter, and intra-bladder pressure as well as the height of the fluid bag if the irrigation fluid demonstrates laminar flow.

This study has the following limitations. We used 0.9% NS as the irrigation fluid for CBI, but different types of fluids, such as antibiotics solutions, may be used in clinical practice for pharmacological treatment purposes, as opposed to achieving hemostasis. Hence, the study could not explore the application of various types of irrigation fluids. Another limitation is that only the minimum and maximum bladder pressures in resting supine position in adults were used for the friction-adjusted Bernoulli’s equation, without considering different bladder pressures observed in other diseases.

## Conclusion

This study aimed to analyze the characteristics of flow during CBI according to the height of the fluid bag and size of indwelling catheter, while considering the temperature of the fluid and intra-bladder pressure, to provide foundational data for CBI. The results showed that at all sizes of the indwelling catheters, flow rate significantly increased with increasing height of irrigation infusion set in both cases using room-temperature fluid (22 °C 0.9% NS) and cold fluid (4 °C 0.9% NS) as well as in cases of minimum (5 cmH_2_O) and maximum (20 cmH_2_O) bladder pressures. When the height of the fluid bag was constant, flow rate was higher with a room-temperature (22 °C) 0.9% NS than with cold (4 °C) NS at all sizes of indwelling catheters. Through the findings of this study, nurses can plan the exchange time of irrigation fluid and the pattern of urinary drainage when performing continuous bladder irrigation. It is also inferred that there may be an advantage in not having to calculate additional material costs for using an infusion pump for patients by determining the hourly injection rate of irrigation fluid based on the height of the infusion set's drop chamber. Based on the results of this study, we present the following recommendations. Subsequent studies should analyze the flow rates of various bladder irrigation fluids used for purposes other than to achieve hemostasis, such as pharmacological treatment. Additionally, it is important to analyze the flow rates of irrigation fluids during CBI while considering varying levels of bladder pressure observed in different diseases and patient states.

## Methods

### Study design

This is a control volume analysis to examine the flow rate of irrigation fluid according to the size of indwelling catheter and the height of the fluid bag in consideration of the temperature of irrigation fluid and intra-bladder pressure during CBI.

### Subject of interpretation

**Figure 3:**
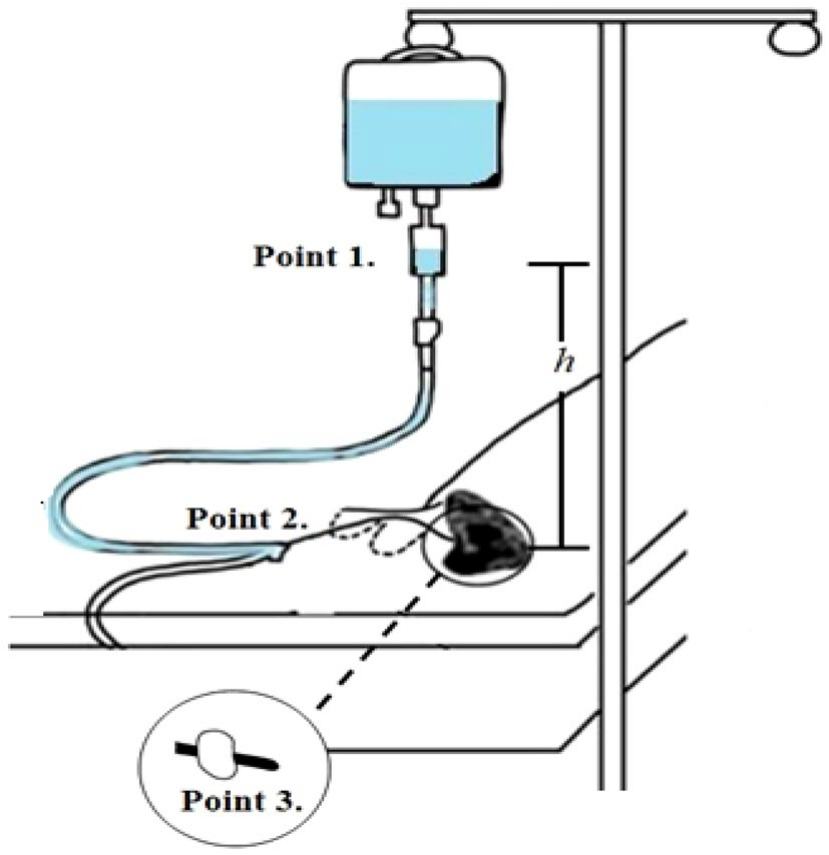
System of continuous bladder irrigation. Point 1: The midpoint of the drip chamber in the irrigation infusion set, Point 2: The irrigation infusion set connects to the indwelling catheter, Point 3:The end point of the indwelling catheter, Irrigation fluid: 22 °C (ρ = 1004.6 kg/m^3^, ν = 0.00102 Pa s), 4 °C (ρ = 1006 kg/m^3^,ν = 0.00163493 Pa s), Intra-bladder pressure at a resting supine position in adult: minimum pressure 5 cmH_2_O (= 490.319 Pa), Maximum pressure 20 cmH_2_O (= 1961.276 Pa), Indwelling catheter (Three-lumen catheter): 18–24Fr, Height of irrigation infusion set: 10 cm from 0 to 120 cm.

The subject of this study is the irrigation infusion sets and indwelling catheters. The irrigation infusion set and indwelling catheter are connected in a straight line, and the end of the indwelling catheter is connected to the bladder. Figure 3 illustrates the subject of interpretation in detail.

#### Irrigation fluid

As the density and viscosity of fluid are altered by temperature^21^, the temperature of the irrigation fluid during CBI alters the flow rate. In this study, we used 0.9% NS as the irrigation fluid and examined two common temperature conditions used for CBI: room temperature (22 °C) and cold (4 °C)^9, 10^.

#### Irrigation infusion set

A general irrigation infusion set (Sewoon Medical Co., Ltd) certified by the Korea Standard was used (KS P ISO 8536-4).

#### Height of irrigation infusion set

The fluid bag changes shape as the volume of irrigation fluid decreases over time. Considering the limitations of examining the flow rate between the spike and drip chamber using a friction-adjusted Bernoulli's equation due to the fluctuating flow in this area, the height of the irrigation infusion set was changed from the height at half the level of the drip chamber to that of the bladder region where the indwelling catheter is inserted, which is the space in which the flow rate is steady.

#### Indwelling catheter

We used (Yushin Medical Co. Ltd) 18Fr, 20Fr, 22Fr, and 24Fr three-lumen catheters, as these are the common sizes used for bladder irrigation.

#### Connection between the irrigation infusion set and the indwelling catheter

In this study, the irrigation infusion set and indwelling catheter were each analyzed using the friction-adjusted Bernoulli's equation. The midpoint of the drip chamber in the irrigation infusion set was set as point 1, and the point where the irrigation infusion set connects to the indwelling catheter was set as point 2. Point 2 strictly refers to the end portion of the irrigation infusion set. In this study, the friction-adjusted Bernoulli's equation applied to the irrigation infusion set and the friction-adjusted Bernoulli's equation applied to the indwelling catheter was linked to analyze the entire target of analysis using a characteristic equation. Therefore, it is important to ensure an appropriate fluid dynamics approach for the boundary between the irrigation infusion set and the indwelling catheter. In terms of pressure, since the irrigation infusion set and the indwelling catheter are physically connected, the pressure at the end point of the irrigation infusion set is equal to that at the starting point of the indwelling catheter. However, velocity is changed at the point where the irrigation infusion set and the indwelling catheter meet, as two tubes with different diameters are connected. In other words, the velocity at the end portion of the irrigation infusion set differs from that at the starting portion of the indwelling catheter. This velocity difference is determined by the continuity equation (mass conservation equation), and the friction-adjusted Bernoulli's equation applied to the irrigation infusion set and the friction-adjusted Bernoulli's equation applied to the indwelling catheter are fluid-dynamically connected through the continuity equation. Finally, the end point of the indwelling catheter that is connected to the bladder was set as point 3.

#### Intra-bladder pressure

The pressure of the bladder into which fluid is flown was set at the minimum pressure (5 cmH_2_O) and maximum pressure (20 cmH_2_O) at a resting supine position in adults^27^.

### Data collection

The study data were collected through a literature review, information provided by the manufacturer, and direct measurements. The specific parameters are described below.

#### Density and viscosity of irrigation fluid

The density and viscosity of the irrigation fluid were obtained from existing literature The density (ρ) and viscosity (μ) of 0.9% NS at 22 °C were 1004.6 kg/m^3^ and 1.020e^−3^ Pa s, respectively, and that of 0.9% NS at 4 °C were 1006.0 kg/m^3^ and 1.635e^−3^ Pa s, respectively^28^.

#### Length and inner diameter of irrigation infusion set

In this study, a KS-certified irrigation infusion set (Sewoon Medical Co., Ltd) was chosen, and the length of the irrigation infusion set was measured by obtaining the length of the drip chamber and tube of irrigation infusion set presented by the KS medical product (KS P ISO 8536-4) details. The length of the drip chamber and tube were 4 cm and 170 cm, respectively. The inner diameters of the irrigation infusion set drip chamber and tube are not provided by KS medical products (KS P ISO 8536-4); therefore, it was measured using a digital caliper (Guilin Guanglu Measuring Instrument Co., Ltd.). The inner diameter of the irrigation infusion set was measured twice using a digital caliper and averaged. The averaged inner diameter of the drip chamber (d_1_) was 13.05 mm, and that of the tube (d_2_) was 1.81 mm.

#### Height from the bladder to the irrigation infusion set

With reference to the height of the fluid bags proposed in previous studies^2, 12^, the height of the irrigation infusion set in this study varied by 10 cm from 0 to 120 cm to analyze the flow rates according to the height.

#### Length and inner diameter of indwelling catheter

The length of the indwelling catheter was taken from the product information provided by the manufacturer (Yushin Medical Co. Ltd). The length of the 18Fr, 20Fr, 22Fr, and 24Fr catheters was set to 40 cm (L_C_). The inner diameter of the indwelling catheter is not provided by the KS medical product information and manufacturer, so it was measured using a digital caliper. The inner diameter of the irrigation port of the indwelling catheter was measured twice using a digital caliper and averaged. The averaged inner diameter of the indwelling catheter (d_3_) was 1.40 mm (18Fr), 1.58 mm (20Fr), 1.64 mm (22Fr), and 1.66 mm (24Fr).

#### Intra-bladder pressure

Intra-bladder pressure at resting supine position in adults (P_3_) was obtained from the literature. The minimum pressure was 490.3 Pa (5 cmH_2_O), and the maximum pressure was 1961 Pa (20 cmH_2_O)^27^.

### Data analysis

#### Assumptions

For the friction-adjusted Bernoulli’s equation used in this study to be valid, the assumptions of incompressible flow, steady state, and no energy transfer with the surroundings must be satisfied for the total analysis domain. The assumption of incompressible flow is satisfied because the fluid used in this study is physiological saline solution in liquid state (at 22 °C or 4 °C), and to exhibit compressibility, the fluid requires velocities close to the speed of sound (approximately 1500 m/s); thus, compressibility can be ignored within the range of velocities for the irrigation fluid entering the fluid bag or bladder. Although the drip chamber in the irrigation infusion set used in this study collects drops of fluid that may lead to an unsteady state, this localized area is negligible compared with the overall analysis domain. Therefore, the unsteady state occurring at the drip chamber has minimal impact on the results of this study, and the assumption of steady state can be considered satisfied. Finally, the energy transfer between the irrigation infusion set and the surroundings is negligible in magnitude. Therefore, the assumptions utilized in the application of the friction-adjusted Bernoulli's equation are considered appropriate for the data analysis and interpretation in this study.

#### Continuity equation

In this study, control volume analysis was performed using the continuity and Bernoulli equations while considering friction based on the assumptions. The continuity equation applied to the analysis domain in this study is shown in Eq. (1).

1 $${\text{A}}_{1} {\text{V}}_{1} = {\text{A}}_{2} {\text{V}}_{2} = {\text{A}}_{3} {\text{V}}_{3}$$

where A and V represent cross-sectional area and area-averaged velocity, respectively. As previously mentioned, subscript 1 refers to half of drip chamber, subscript 2 refers to connection between the irrigation infusion set and indwelling catheter (end of irrigation infusion set). Subscript 3 is the end of the indwelling catheter. A_1_V_1_, A_2_V_2_, A_3_V_3_ are the volumetric flow rates at half point of drip chamber, end of the irrigation infusion set, and end of the indwelling catheter, respectively. The velocity of fluid at each point can be calculated based on the inner diameter of the tube at each point.

#### Friction-adjusted Bernoulli’s equation

The Bernoulli equations that consider friction and are applied to the target of analysis are as follows. The friction-adjusted Bernoulli equations applied between point 1 and point 2, and between point 2 and point 3 are represented by Eqs. (2) and (3), respectively. By adding Eqs. (2) and (3), the friction-adjusted Bernoulli's equation applied between point 1 and point 3, which represents the beginning and end of the entire target of analysis, can be expressed, as given in Eq. (4).

2 $$\frac{{{\text{P}}_{1} }}{{{\rho g}}} + \frac{{{\alpha V}_{1}^{2} }}{{2{\text{g}}}} + {\text{z}}_{1} = \frac{{{\text{P}}_{2} }}{{{\rho g}}} + \frac{{{\alpha V}_{2}^{2} }}{{2{\text{g}}}} + {\text{z}}_{2} + {\text{h}}_{{{\text{f}},1{-}2}}$$

3 $$\frac{{{\text{P}}_{2} }}{{{\rho g}}} + \frac{{{\alpha V}_{2}^{2} }}{{2{\text{g}}}} + {\text{z}}_{2} = \frac{{{\text{P}}_{3} }}{{{\rho g}}} + \frac{{{\alpha V}_{3}^{2} }}{{2{\text{g}}}} + {\text{z}}_{3} + {\text{h}}_{{{\text{f}},2{-}3}}$$

4 $$\frac{{{\text{P}}_{1} }}{{{\rho g}}} + \frac{{{\alpha V}_{1}^{2} }}{{2{\text{g}}}} + {\text{z}}_{1} = \frac{{{\text{P}}_{3} }}{{{\rho g}}} + \frac{{{\alpha V}_{3}^{2} }}{{2{\text{g}}}} + {\text{z}}_{3} + {\text{h}}_{{{\text{f}},1{-}3}}$$

where P, ρ, g, z and h_f_ represent pressure, density, gravity constant, height and head loss due to the friction, respectively. The term α is the kinetic energy correction factor, having a value of about 2.0 for fully developed laminar pipe flow.

#### Characteristic equation

In this study, a characteristic equation was derived by simultaneously solving the continuity equation and the friction-adjusted Bernoulli's equation to investigate the flow characteristics of the irrigation fluid entering the bladder based on factors such as fluid temperature, bladder pressure, height of the irrigation infusion set, and size of the indwelling catheter. The resulting characteristic equation represents the relationship between the temperature of the irrigation fluid, bladder pressure, height of the irrigation infusion set, size of the indwelling catheter, and the flow rate of the irrigation fluid entering the bladder and was derived as follows:

The total head loss of the entire target of analysis can be calculated by summing up the head losses in the irrigation infusion set and indwelling catheter, as given in Eq. (5).

5 $${\text{h}}_{{{\text{f}},1{-}3}} = {\text{h}}_{{{\text{f}},1{-}2}} + {\text{h}}_{{{\text{f}},2{-}3}} = {\text{f}}_{{{\text{IS}}}} \frac{{{\text{L}}_{{{\text{IS}}}} }}{{{\text{d}}_{{{\text{IS}}}} }}\frac{{{\text{V}}_{{{\text{IS}}}}^{2} }}{{2{\text{g}}}} + {\text{f}}_{{\text{C}}} \frac{{{\text{L}}_{{\text{C}}} }}{{{\text{d}}_{{\text{C}}} }}\frac{{{\text{V}}_{{\text{C}}}^{2} }}{{2{\text{g}}}}$$

Subscript IS represents the irrigation infusion set, and f_IS_ is the Darcy friction factor of the irrigation infusion set. L_IS_ is the length of the fluid tube, while d_IS_ is the diameter of the fluid tube. V_IS_ is the area-average velocity of the fluid flowing through the tube. Subscript C refers to the indwelling catheter, and f_C_ is the Darcy friction factor of the indwelling catheter, and L_C_ is the length of the indwelling catheter. d_C_ is the diameter of the indwelling catheter, and V_C_ is the area-average velocity of the fluid flowing through the indwelling catheter.

Additional head loss occurs at the zones at which the intra-tube diameter changes between point 1 and point 3 (between drip chamber and fluid tube, and between fluid tube and indwelling catheter). However, the length of these zones is negligible compared with the entire system of analysis, so we did not consider head loss in these zones. If the head loss in the zones of diameter change in Eq. (5) is negligible, Eq. (6) can be used.

6 $${\text{h}}_{{{\text{f}},1{-}3}} = {\text{h}}_{{{\text{f}},1{-}2}} + {\text{h}}_{{{\text{f}},2{-}3}} = {\text{f}}_{{{\text{IS}}}} \frac{{{\text{L}}_{{{\text{IS}}}} }}{{{\text{d}}_{2} }}\frac{{{\text{V}}_{2}^{2} }}{{2{\text{g}}}} + {\text{f}}_{{\text{C}}} \frac{{{\text{L}}_{{\text{C}}} }}{{{\text{d}}_{3} }}\frac{{{\text{V}}_{3}^{2} }}{{2{\text{g}}}}$$

Using the definition of the Darcy friction factor for laminar flow (f = 64μ/ρVd Eq. (6) can be expressed as Eq. (7). Where μ represents the dynamic viscosity. In the case of turbulent flow, the Darcy friction factor is expressed differently. However, the Re for the irrigation infusion set and the indwelling catheter confirmed a laminar flow.

7 $${\text{h}}_{{{\text{f}},1{-}3}} = {\text{h}}_{{{\text{f}},1{-}2}} + {\text{h}}_{{{\text{f}},2{-}3}} = \frac{{64{\upmu }}}{{{\rho V}_{2} {\text{d}}_{2} }}\frac{{{\text{L}}_{{{\text{IS}}}} }}{{{\text{d}}_{2} }}\frac{{{\text{V}}_{2}^{2} }}{{2{\text{g}}}} + \frac{{64{\upmu }}}{{{\rho V}_{3} {\text{d}}_{3} }}\frac{{{\text{L}}_{{\text{C}}} }}{{{\text{d}}_{3} }}\frac{{{\text{V}}_{3}^{2} }}{{2{\text{g}}}}$$

From the continuity equation (Eq. 1), V_2_ can be expressed as a function of V_3_ in the following form:

8 $${\text{V}}_{2} = \frac{{{\text{d}}_{3}^{2} }}{{{\text{d}}_{2}^{2} }}{\text{V}}_{3}$$

By entering Eq. (8) into Eq. (7), the head losses, h_f,1–3_, can be expressed as a function of V_3_, as shown in Eq. (9):

9 $${\text{h}}_{{{\text{f}},1{-}3}} = {\text{kV}}_{3}$$

Here, k is a constant that can be expressed based on the geometric information of the drip chamber, the tubing in the irrigation infusion set, and the indwelling catheter:

10 $${\text{k}} = \frac{{32{\mu L}_{{{\text{IS}}}} }}{{{\rho d}_{2}^{2} {\text{g}}}}\frac{{{\text{d}}_{3}^{2} }}{{{\text{d}}_{2}^{2} }} + \frac{{32{\mu L}_{{\text{C}}} }}{{{\rho d}_{3}^{2} {\text{g}}}}$$

Equation (4), which is the Bernoulli equation of friction from point 1 (1/2 point of the drip chamber) to point 3 (end of indwelling catheter), can be expressed in the form of Eq. (11) as follows:

11 $$\frac{{{\text{P}}_{1} }}{{{\rho g}}} + \frac{{2{\text{V}}_{1}^{2} }}{{2{\text{g}}}} + {\text{z}}_{1} = \frac{{{\text{P}}_{3} }}{{{\rho g}}} + \frac{{2{\text{V}}_{3}^{2} }}{{2{\text{g}}}} + {\text{z}}_{3} + {\text{kV}}_{3}$$

At point 1 (midpoint in drip chamber), the pressure is approximately zero as the drip chamber is sealed off from the atmosphere and exists in a vacuum-like state with only vapor pressure. If the end of the indwelling catheter (point 3) is set as the reference point for height measurement, z_3_ = 0, and h represents the vertical distance (difference in height in the opposite direction of gravity) from the position of the catheter to the midpoint of drip chamber.

Using the definition of the Darcy friction factor for laminar flow (f = 64/Re), Eq. (6) can be expressed as Eq. (7).

Furthermore, using the continuity equation (Eq. 1), ${\text{V}}_{1}^{2} = \left( {{\text{d}}_{3}^{4} /{\text{d}}_{1}^{4} } \right){\text{V}}_{3}^{2}$. Thus, Eq. (11) can be expressed as a quadratic equation of V_3_ as shown in Eq. (12):

12 $$\frac{1}{{\text{g}}}\left( {\frac{{{\text{d}}_{3}^{4} }}{{{\text{d}}_{1}^{4} }} - 1} \right){\text{V}}_{3}^{2} - {\text{kV}}_{3} + \left( {{\text{h}} - \frac{{{\text{P}}_{3} }}{{{\rho g}}}} \right) = 0$$

Thus, we can analyze the relationship among the geometric shape of the irrigation infusion set and the indwelling catheter, bladder pressure (P_3_), the height from the end of the catheter to the midpoint of the drip chamber (h), and the flow rate of the irrigation fluid by obtaining the solution to the quadratic equation of the characteristic equation (Eq. 12).

#### Reynolds number

In this study, the validity of the laminar flow assumption was assessed by calculating the Reynolds number (Re).

13 $${\text{Re}} = \frac{{\uprho {\text{VD}}}}{{\upmu }}$$

### Supplementary Information

Supplementary Information.

## Data Availability

The datasets used and/or analyzed during the current study available from the corresponding author on reasonable request.
