# Supplementary material for: Analysis of flow rate of continuous bladder irrigation according to the height of the irrigation infusion set
Source: Sci Rep. 2023 Nov 12;13:19715. doi: 10.1038/s41598-023-47198-2 (PMC10641072; doi:10.1038/s41598-023-47198-2)
Supplement: Supplementary file 1 — Supplementary Information. [file 41598_2023_47198_MOESM1_ESM.docx]

| **Supplement 1**. Comparison of flow rate according to the size of indwelling catheter and height of irrigation infusion set | | | | | | | | | |
| --- | --- | --- | --- | --- | --- | --- | --- | --- | --- |
| **Height (m)** | **Intra-bladder pressure (cmH_2_O)** | **Flow rate (cc/hr)** | | | | | | | |
|  |  | **4 ℃** | | | | **22 ℃** | | | |
|  |  | **18Fr** | **20Fr** | **22Fr** | **24Fr** | **18Fr** | **20Fr** | **22Fr** | **24Fr** |
| **0** | **5** | -100.8 | -118.8 | -122.4 | -126 | -162.0 | -190.8 | -198.0 | -198.0 |
| **0.1** |  | 100.8 | 118.8 | 126.0 | 126 | 162.0 | 190.8 | 198.0 | 201.6 |
| **0.2** |  | 302.4 | 356.4 | 370.8 | 374.4 | 482.4 | 568.8 | 594.0 | 601.2 |
| **0.3** |  | 504.0 | 594.0 | 619.2 | 626.4 | 799.2 | 943.2 | 982.8 | 997.2 |
| **0.4** |  | 702.0 | 828.0 | 864.0 | 874.8 | 1116.0 | 1317.6 | 1375.2 | 1389.6 |
| **0.5** |  | 903.6 | 1065.6 | 1108.8 | 1123.2 | 1432.8 | 1688.4 | 1760.4 | 1782.0 |
| **0.6** |  | 1101.6 | 1299.6 | 1353.6 | 1371.6 | 1742.4 | 2059.2 | 2145.6 | 2174.4 |
| **0.7** |  | 1299.6 | 1533.6 | 1598.4 | 1616.4 | 2052.0 | 2426.4 | 2530.8 | 2563.2 |
| **0.8** |  | 1497.6 | 1767.6 | 1839.6 | 1864.8 | 2361.6 | 2793.6 | 2912.4 | 2948.4 |
| **0.9** |  | 1695.6 | 2001.6 | 2084.4 | 2109.6 | 2667.6 | 3157.2 | 3290.4 | 3333.6 |
| **1.0** |  | 1893.6 | 2232.0 | 2325.6 | 2358.0 | 2973.6 | 3517.2 | 3668.4 | 3715.2 |
| **1.1** |  | 2088.0 | 2466.0 | 2570.4 | 2602.8 | 3276.0 | 3877.2 | 4042.8 | 4096.8 |
| **1.2** |  | 2286.0 | 2696.4 | 2811.6 | 2847.6 | 3574.8 | 4233.6 | 4417.2 | 4474.8 |
| **0** | **20** | -403.2 | -471.6 | -493.2 | -500.4 | -648.0 | -763.2 | -792.0 | -802.8 |
| **0.1** |  | -198.0 | -234.0 | -244.8 | -248.4 | -320.4 | -378.0 | -392.4 | -399.6 |
| **0.2** |  | 3.6 | 3.6 | 3.6 | 3.6 | 3.6 | 3.6 | 3.6 | 3.6 |
| **0.3** |  | 205.2 | 241.2 | 248.4 | 252.0 | 324.0 | 381.6 | 399.6 | 403.2 |
| **0.4** |  | 403.2 | 475.2 | 496.8 | 504.0 | 644.4 | 759.6 | 792.0 | 802.8 |
| **0.5** |  | 604.8 | 712.8 | 741.6 | 752.4 | 961.2 | 1134.0 | 1180.8 | 1195.2 |
| **0.6** |  | 806.4 | 950.4 | 986.4 | 1000.8 | 1278.0 | 1508.4 | 1569.6 | 1591.2 |
| **0.7** |  | 1004.4 | 1184.4 | 1234.8 | 1249.2 | 1591.2 | 1875.6 | 1958.4 | 1980.0 |
| **0.8** |  | 1202.4 | 1418.4 | 1476.0 | 1497.6 | 1900.8 | 2246.4 | 2340.0 | 2372.4 |
| **0.9** |  | 1400.4 | 1652.4 | 1720.8 | 1742.4 | 2210.4 | 2613.6 | 2725.2 | 2757.6 |
| **1.0** |  | 1598.4 | 1886.4 | 1965.6 | 1990.8 | 2516.4 | 2977.2 | 3103.2 | 3142.8 |
| **1.1** |  | 1796.4 | 2120.4 | 2206.8 | 2235.6 | 2822.4 | 3340.8 | 3481.2 | 3528.0 |
| **1.2** |  | 1990.8 | 2350.8 | 2451.6 | 2480.4 | 3124.8 | 3700.8 | 3859.2 | 3909.6 |
